# Role of Antioxidant Vitamins in Oral Submucous Fibrosis: A Narrative Review

**DOI:** 10.7759/cureus.59596

**Published:** 2024-05-03

**Authors:** Ravikant V Sune, Rahul R Bhowate, Vidya K Lohe, Suwarna B Dangore-Khasbage

**Affiliations:** 1 Oral Medicine and Radiology, Sharad Pawar Dental College and Hospital, Datta Meghe Institute of Higher Education and Research, Wardha, IND

**Keywords:** oxidant damage and antioxidants, vitamin e deficiency, vitamin c deficiency, vitamin a deficiency, oral submucous fibrosis (osmf/osf)

## Abstract

Oral submucous fibrosis (OSMF) has a high rate of malignant transformation and is an insidious chronic inflammatory disease. Though this disorder seems to be multifactorial in origin, betel quid chewing appears to be the main etiologic factor. Various treatment strategies have been attempted but none proven to cure the disorder because of its multimodal pathogenesis. Reactive oxygen species (ROS) appear to have a role in cancer formation. As OSMF is an oral premalignant disorder and found to be associated with carcinogens like areca nut and tobacco, it is believed to have some relationship with ROS. Tissue damage due to ROS along with other mechanisms may result in the complex pathophysiology of OSMF. The antioxidant system in the body helps to prevent damage caused by highly reactive ROS and helps in the repair of tissues. To study the levels of oxidative stress and antioxidant vitamins in OSMF condition, the present review was done. We carried out a thorough literature search to identify original reports and studies determining the status of oxidative stress and antioxidant vitamins in OSMF condition using several databases including Google Scholar, PubMed, and Scopus. Our review observed that the oxidative stress increased in the condition of OSMF as shown by an increase in malonaldehyde (MDA) and a decrease in antioxidant vitamins like vitamin A, vitamin C, and vitamin E. Also, after the intake of antioxidant vitamins, there was symptomatic improvement in OSMF patients. With the help of identifying oxidative stress and antioxidant status, we can assess the clinical stage of OSMF and can develop a comprehensive treatment plan.

## Introduction and background

Oral submucous fibrosis (OSMF) was initially documented as "atropica idiopathica mucosae oris" by Schwartz in 1952. Later, described by Jens J. Pindborg in 1966, OSMF is a chronic, insidious disorder affecting the oral and sometimes pharyngeal mucosa associated with inflammatory reaction in the sub-epithelium and occasionally vesicle formation which is followed by fibrosis in the lamina propria with atrophy of the epithelium resulting in stiffened oral mucosa, related trismus, and difficulty in eating [1]. The majority of the cases occur in India and in people who migrated from the Indian subcontinent. It is estimated that around 2.5 million people are affected by OSMF in 1996 worldwide [2]. But the cases are increasing, and recent data estimates that in India alone, more than five million people are affected [3]. The rate of transformation of OSMF into malignancy was observed to be 7.6% in a 17-year follow-up study [4]. This may vary from 1.9% to 9% according to the diagnostic criteria used and follow-up duration in various studies [4-8].

The etiology of OSMF seems to be multifactorial. The main etiologic factor is the chewing habit of areca nut and its flavored preparations with or without tobacco. Other contributory factors are nutritional deficiency, anemia, and genetic and immunologic predisposition. Areca nut alkaloids arecoline, arecaidine, guvacine, and guvacoline especially arecoline have been found in various studies to have a significant role in OSMF pathogenesis. Alkaloids are known for their stimulation activity of collagen production. Flavonoids (tannins and catechins) inhibit the degradation of collagen by collagenase. Also, areca nut contains a high amount of copper which further increases the cross-linking of collagen fibers by enhancement of the lysyl oxidase pathway [9-15]. Numerous therapeutic strategies have been tried in the past and are mainly directed against the signs and symptoms of the OSMF disorder. Since its description as a potentially malignant disorder for almost seven decades, no significant improvement in treatment is present to cure the disease because of the multifactorial pathogenesis of OSMF [16].

The generation of reactive oxygen species (ROS) has been known to be associated with the process of carcinogenesis as shown by many epidemiological studies [17]. These ROS initiate lipid peroxidation and cause DNA damage [18]. OSMF is an oral premalignant disorder with a substantial rate of malignant transformation. Also, OSMF is associated with carcinogens like areca nut and tobacco. Areca nut contents such as arecoline, tannins, and copper can increase ROS in oral tissues. In view of this, it is considered that OSMF has some relationship with ROS. So, this review was done to evaluate the levels of oxidative stress and antioxidant vitamins in patients with OSMF and the significance of antioxidant vitamins in the treatment of OSMF.

## Review

Methodology

To search the original reports and studies determining the levels of oxidative stress and antioxidant vitamins in OSMF patients and the significance of antioxidant vitamins in their treatment, a thorough literature search was done using several databases including Google Scholar, PubMed, and Scopus. Various medical subject headings (MeSH) phrases and keywords utilized for the literature search included "oral submucous fibrosis," "vitamin C deficiency," "vitamin E deficiency," "vitamin A deficiency," "oxidative stress," and "antioxidants." We undertook the literature search in August 2023. All investigative and treatment studies pertaining to oxidative stress and antioxidant vitamins in OSMF were included in the review. Individual case reports and studies with antioxidants not including vitamins were excluded from the present review. Also, studies available in languages other than English were excluded. Studies with positive results showing the importance of antioxidants are more often available than negative results which may have been the limitation of this review. Also, studies published only in the English language were searched which may have missed the relevant data in other languages and may be the limitation of this review.

Results

Oxidative stress is considered by determining lipid peroxidation products caused by ROS such as malonaldehyde (MDA), and the primary antioxidant vitamins considered are vitamin A, vitamin C, and vitamin E. A total of 19 studies determining the levels of oxidative stress and antioxidant vitamins in OSMF patients and the significance of antioxidant vitamins in their treatment were included in the present review. Eleven studies were based on biochemical analysis before the treatment between the OSMF patients and control group, and seven studies were related to clinical analysis before and after the treatment in OSMF patients. One study conducted both biochemical analysis and clinical analysis before and after the treatment in OSMF patients. β-Carotene is considered a provitamin of vitamin A. Among carotenoids, β-carotene is mainly transformed into vitamin A in our body. So, studies related to β-carotene were also included in the present review. The primary outcome of studies conducting biochemical analysis was that the level of oxidative stress increased in patients of OSMF as shown by an increase in MDA and a decrease in antioxidants like superoxide dismutase (SOD), vitamin A, vitamin C, and vitamin E in OSMF patients. The major outcome of studies related to clinical analysis after treatment is that after the intake of antioxidants, there was symptomatic improvement in OSMF patients. Table 1 shows the list of studies evaluating oxidative stress and antioxidant vitamins in OSMF.

**Table 1:** List of studies evaluating oxidative stress and antioxidant vitamins in OSMF MDA: malonaldehyde; SOD: superoxide dismutase; Cu: copper; Fe: iron; Zn: zinc; Mg: magnesium; Ca: calcium; 8-OHdG: 8-hydroxy-deoxyguanosine; Se: selenium; Mn: manganese

Authors	Type of analysis	Ingredients studied
Gupta et al. [19]	Biochemical analysis and clinical analysis	MDA, β-carotene, vitamin E, SOD
Borle and Borle [20]	Clinical analysis	Vitamin A
Maher et al. [21]	Clinical analysis	Multiple micronutrients and minerals (vitamin A, vitamin C, vitamin E, vitamin B complex, vitamin D, Fe, Zn, Cu, Ca, Mg, and others)
Metkari et al. [22]	Biochemical analysis	MDA, SOD, vitamin A
Rai et al. [23]	Biochemical analysis	Vitamin C, vitamin E, MDA, 8-OHdG, curcumin
Dhariwal et al. [24]	Clinical analysis	Vitamin A, zinc
Shetty et al. [25]	Biochemical analysis	Vitamin C
Bose et al. [26]	Biochemical analysis	Total antioxidant activity, vitamin C, vitamin E, β-carotene, glutathione
Aravindh et al. [27]	Biochemical analysis	β-Carotene, vitamin C, vitamin E
Mehrotra et al. [28]	Clinical analysis	Multi-nutrient drugs (vitamin A, vitamin C, vitamin B complex, Se, Zn, Fe, Mn, Cu)
Guruprasad et al. [29]	Biochemical analysis	Vitamin C, iron
Nayak et al. [30]	Clinical analysis	Vitamin E, lycopene
Nallapu et al. [31]	Clinical analysis	Vitamin E, dexamethasone, hyaluronidase, lignocaine
Rai et al. [32]	Biochemical analysis	MDA, SOD, vitamin E, β-carotene
Bhat et al. [33]	Biochemical analysis	Vitamin C
Bhalerao et al. [34]	Biochemical analysis	Vitamin C, iron
Kumar et al. [35]	Clinical analysis	Vitamin E, lycopene
Jain et al. [36]	Biochemical analysis	Vitamin A, vitamin E
Oswal et al. [37]	Biochemical analysis	MDA, vitamin E, β-carotene
Nilesh et al. [38]	Clinical analysis	Multi-drug therapy (β-carotene, lycopene, Zn, Cu, Se, alpha-lipoic acid, alpha-tocopheryl acetate)
Shah et al. [39]	Clinical analysis	Vitamin E, pentoxifylline, triamcinolone

Discussion

ROS

An atom or a molecule containing an unpaired electron in its valency orbit is unstable, and it can remain in such form and is known as a free radical. Although oxygen is utilized by the body as a metabolic fuel, a significant fraction of it gets incompletely reduced and forms oxygen free radicals and their byproducts. They are highly reactive and toxic and can cause tissue damage. Collectively, these free radicals of oxygen and their derivatives are known as ROS. ROS include superoxides, hydrogen peroxides, and hydroxyl radicals. A highly reactive hydroxyl radical is capable of initiating toxic reactions against tissues and cells such as DNA damage and lipid peroxidation [40].

Mechanism of Action of ROS and Antioxidants

ROS are toxic and can cause damage to tissue by lipid peroxidation (through the activation of lipo-oxygenase and cyclo-oxygenase pathways), DNA damage, protein damage such as proteoglycans and hyaluronic acid, oxidation of some of the important enzymes such as antitrypsin antiprotease, increase in pro-inflammatory cytokine release by monocytes and macrophages through the stimulation of nuclear factor, and depletion of intracellular thiol compounds. Antioxidants counteract ROS effects by facilitating the repair of damage caused by ROS, preventing the generation of free radicals, intercepting the actions of free radicals, and creating a favorable tissue environment [41,42].

Oxidative Stress and Antioxidants

**Figure 1:**
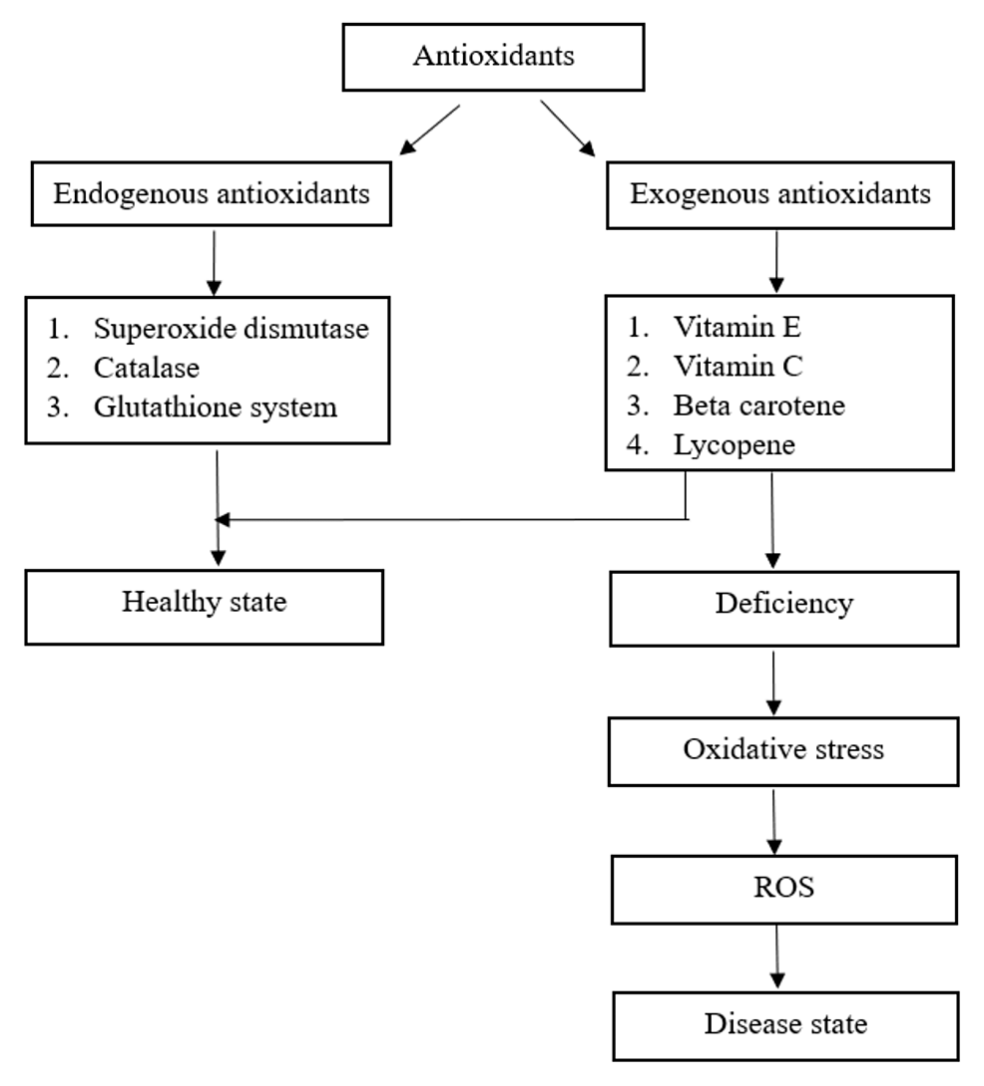
Oxidative stress and antioxidants ROS: reactive oxygen species

When there is a shift in pro- and antioxidant balance more towards pro-oxidant, oxidative stress follows. Oxidative stress will cause damage to cells and tissues when ROS exceeds the antioxidant protective mechanism of the body. The antioxidants that originate within the body are known as endogenous antioxidants. These physiologic antioxidants on their own are not sufficient to remove ROS; thus, antioxidants need to be supplied from outside the body. Antioxidants administered as nutrients in the form of food or supplements are exogenous antioxidants. Endogenous and exogenous antioxidants act together to reduce ROS-dependent damage. Figure 1 shows the relationship between antioxidants and oxidative stress. The primary endogenous antioxidants are SOD, catalase, and the glutathione system. Among exogenous antioxidants, the important are vitamin C and vitamin E. Another group of exogenous antioxidants that have a considerable effect are carotenoids, especially β-carotene and lycopene. Epidemiological studies in their research have observed that diets containing green leafy vegetables and fruits result in a decreased risk of cancer. These vegetables contain antioxidants, and certain elements in the vegetables may have the capacity to induce antioxidant enzymes [40].

Studies Evaluating Oxidative Stress and Antioxidant Vitamins in OSMF

Gupta et al., in their study, found significantly increased MDA, a lipid peroxidation product, and reduced plasma levels of β-carotene and vitamin E in OSMF patients in comparison with controls which implicates that oxidative stress is increased and the antioxidant vitamins are decreased in OSMF. In the same study after six weeks of treatment with oral β-carotene and vitamin E supplements, reduced levels of MDA and an increase in β-carotene and vitamin E with clinical improvement with respect to mouth opening and tongue protrusion were noted in OSMF patients [19]. This study showed that there can be involvement of ROS in the etiopathogenesis of OSMF and antioxidant vitamins can have a protective role. Borle and Borle, in their study, showed that vitamin A could cause symptomatic improvement but trismus was unaffected in OSMF cases [20]. Maher et al., in their study with multiple micronutrients consisting of multivitamins including vitamins A, C, E, B complex, and D and multi-minerals such as iron, zinc, copper, calcium, Mg, and others in the treatment of OSMF for a period of one to three years, showed clinical improvement significantly in terms of burning sensation, inability to tolerate spicy food, and reduced mouth opening [21]. This study showed a beneficial response to multiple micronutrients in OSMF patients. However, this study did not give any specificity to a particular micronutrient but determined the role of multiple micronutrients in treatment.

Metkari et al. found that lipid peroxidation product MDA was increased significantly in the sera of patients with OSMF, whereas SOD and vitamin A were decreased significantly. They also found that MDA increased significantly as the severity of the disease increased clinically [22]. This shows that lipid peroxidation product determines the severity of disease which in turn reflects the extent of tissue injury and the oxidative stress. On the contrary, the levels of SOD and vitamin A decreased significantly as the severity of disease increased. This decrease can be due to the result of combating excessive oxidative stress in circulation or by utilization in affected tissues. Rai et al., in their study, noted decreased salivary and serum vitamin E and C levels and increased salivary and serum 8-hydroxydeoxyguanosine (8-OHdG) and MDA levels in OSMF patients as compared to controls. On curcumin intake, in OSMF patients, there were an increase in vitamin E and vitamin C and a decrease in 8-OHdG and MDA, and the authors concluded that curcumin acts by increasing vitamin E and vitamin C levels and inhibiting peroxidation of lipids and injury to DNA [23]. Dhariwal et al. observed that when zinc acetate is administered along with vitamin A supplement, there were a reduction in burning sensation and an increase in mouth opening and epithelial thickness in OSMF patients; however, it needs to be coupled with stoppage of areca nut chewing habit [24].

Serum and salivary vitamin C levels of patients with OSMF were significantly decreased as found by Shetty et al. in their study. The same study demonstrated vitamin C levels decreased significantly as the histopathological grade of the disease increased [25]. Utilization for collagen synthesis may have been the cause for this reduction. Thus, vitamin C levels can be a valuable indicator of the progression of OSMF. In a study by Bose et al., the levels of antioxidant vitamin A, vitamin E, vitamin C, and glutathione were reduced with the total antioxidant activity hampered in OSMF patients [26]. Their results showed that tobacco or areca quid is responsible for oxidative stress in the causation of OSMF disorder. In a study by Aravindh et al., micronutrient β-carotene, vitamin C, and vitamin E levels were significantly higher in the normal group as compared to OSMF and oral cancer groups. Also, more significantly, decreases in the levels of antioxidant β-carotene, vitamin C, and vitamin E were present in oral cancer patients than in OSMF patients which suggests that an increase in oxidative stress has a role in the transformation of OSMF into cancer of the oral cavity [27]. Mehrotra et al. gave multi-nutrient drugs (vitamin A, vitamin C, vitamin B complex, Se, Zn, Fe, Mn, Cu) to OSMF patients for a period of two to three months and found improvement in burning sensation, repeated painful ulcerations, and stiffness of oral tissue. Improvement in trismus occurred in some cases [28]. In their study, antioxidant therapy was especially advantageous for the early stages of OSMF, and they advised to use antioxidant therapy regularly in OSMF patients. Guruprasad et al. found significantly decreased vitamin C levels in OSMF patients [29]. They made a remark that chemical thermal and/or mechanical injury caused by betel nut chewing along with vitamin C and iron deficiency may cause OSMF and the therapeutic use of vitamin C along with iron can be advised in such cases.

Nayak et al., in their study for the treatment of OSMF patients, used lycopene in group I, vitamin E with lycopene in group II, and placebo drug in group III. They observed that results were more favorable when vitamin E was used with lycopene than with only lycopene or placebo group. Results were more favorable in terms of improvement in mouth opening, burning sensation, and erythematous areas/ulcerations/erosions [30]. Nallapu et al., in their study, used routine treatment of intralesional dexamethasone, hyaluronidase, and lignocaine in one group of OSMF, while the other group received in addition vitamin E capsules. The group with vitamin E showed additional improvement in the extent of mouth opening, tongue protrusion, burning sensation, paleness of oral mucosa, pigmentation, and vesiculation/ulceration [31]. This study showed a significant role of vitamin E in the improvement of OSMF. Rai et al. investigated that MDA level gradually increased and SOD level gradually decreased from normal to OSMF to oral cancer patients and the difference was significantly high. Significantly decreased vitamin E and β-carotene levels were found in patients with OSMF and oral cancer as compared to healthy controls [32]. This study showed that both enzymatic antioxidants and non-enzymatic antioxidant vitamins get deranged indicating oxidative stress increased in OSMF and their values can be used as a valuable tool to indicate the progression of disease. Bhat et al. observed that salivary and serum vitamin C levels were decreased significantly in oral precancer and cancer patients when compared to healthy normal subjects which determines the protective role of vitamin C against lipid peroxidation due to free radicals formed [33].

Bhalerao et al., in their study, found significantly decreased levels of vitamin C and iron in the serum and saliva of OSMF patients in comparison with betel nut habitual and healthy control subjects which caused the authors to believe that vitamin C and iron deficiency are crucial in the pathogenesis of OSMF, but they also suggested that betel nut chewers are at an increased risk for OSMF as their levels of iron and vitamin C were lower than that of healthy people [34]. Kumar et al., in their study with vitamin E and lycopene administration in OSMF patients for three months, found that when vitamin E was used in combination with lycopene, it showed further improvement in mouth opening and burning sensation in OSMF patients than when lycopene was used alone which proves the efficacy of vitamin E in OSMF [35]. Jain et al. observed decreased vitamin A and vitamin E levels in patients with OSMF than controls though the difference was not statistically significant. Also, their levels decreased with the increase in clinical grades of OSMF, but no statistical difference was present between the clinical grades [36]. Though the authors could not find a significant deficiency of antioxidant vitamins in OSMF patients, the variation may be due to geographic or population variance, and more accurate methods of investigation and larger sample size studies are needed to evaluate the antioxidant status in OSMF. Oswal et al. noted higher serum MDA levels and reduced β-carotene and vitamin E in the serum of patients with both OSMF and oral squamous cell carcinoma (OSCC) [37].

Nilesh et al. conducted a multidrug study for the treatment of OSMF. The authors used the combination of lycopene, β-carotene which is pro-vitamin A, zinc, copper, selenium, alpha-lipoic acid, and alpha-tocopheryl acetate which is a form of vitamin E in the management of OSMF. They observed improvement in mouth opening and burning sensation in OSMF patients [38]. The efficiency of the particular drug is difficult to predict from this study, but combination therapy was effective. In a clinical trial conducted by Shah et al. in treating stage 2 and 3 OSMF patients, one group received intralesional triamcinolone and the other oral pentoxifylline and vitamin E, and significant improvement in mouth opening in both groups post-treatment was noted. The authors added antioxidant vitamin E in their study with the understanding that oxidative stress has a role in the progression of OSMF [39]. There is increasing evidence in the literature that oxidative stress plays a crucial role in the pathogenesis of OSMF and possibly its malignant transformation [43,44]. Antioxidant supplements overall are associated with clinical improvement and have a therapeutic potential in OSMF [43].

In summary, OSMF is a chronic disorder with a high rate of malignant transformation. Because of its complex pathophysiology, significant improvement in treatment has not been achieved to cure this disorder. The present review highlighted that the OSMF patients consistently showed increased levels of oxidative stress biomarkers such as MDA and 8-OHdG as determined by various studies included in this review. Also, endogenous antioxidant enzymes such as serum SOD and exogenous antioxidants such as vitamins A, C, and E were decreased. So, it appears that this increased oxidative stress has a crucial role in OSMF pathogenesis and the transformation of OSMF into malignancy. These biomarkers can be used for diagnostic, prognostic, and therapeutic purposes. This review also highlighted the fact that antioxidant vitamin supplementation results in the improvement of signs and symptoms of OSMF such as burning sensation, mouth opening, and tongue protrusion and may be used as chemopreventive agents reducing oxidative stress and preventing the transformation of OSMF into malignancy.

## Conclusions

There are an increase in MDA levels and a decrease in antioxidant vitamin levels such as vitamin C, vitamin E, and vitamin A in OSMF patients as found in various studies in the literature which suggests that oxidative stress increases and the antioxidant status of the body decreases in OSMF. ROS which causes oxidative stress thus has been implicated in the pathogenesis of this premalignant disorder. Monitoring the levels of antioxidant vitamins can be useful biomarkers to assess the progression of the disease, and accordingly, a comprehensive treatment plan can be framed. Administration of antioxidant vitamins helps in clinical improvement and reduces the risk for malignant transformation of OSMF. Prospective clinical trials are required to estimate the doses and duration of therapy of antioxidant vitamins. Also, follow-up studies for longer durations are required to specify the usefulness of the particular antioxidant vitamins.
